# Distinct molecular subtypes of gastric cancer: from Laurén to molecular pathology

**DOI:** 10.18632/oncotarget.24827

**Published:** 2018-04-10

**Authors:** Magdalena Cisło, Agata Anna Filip, George Johan Arnold Offerhaus, Bogumiła Ciseł, Karol Rawicz-Pruszyński, Małgorzata Skierucha, Wojciech Piotr Polkowski

**Affiliations:** ^1^ Department of Surgical Oncology, Medical University of Lublin, Lublin, Poland; ^2^ Department of Cancer Genetics and Cytogenetics Laboratory, Medical University of Lublin, Lublin, Poland; ^3^ Department of Pathology, University Medical Centre Utrecht, Utrecht, The Netherlands; ^4^ Department of Human Anatomy, Medical University of Lublin, Lublin, Poland

**Keywords:** stomach neoplasm, pathology, molecular therapeutics, surgical oncology

## Abstract

In Western countries the majority of gastric cancers (GC) are usually diagnosed in advanced stages reporting a 5-year survival rate of only 26%. The Laurén classification of GC was most widely used in clinical practice since it reflects GC morphology, epidemiology, tumor biology, clinical management and outcome. Despite the initial promise of individualizing antitumor treatment, the management of GC still remains relatively broad and general. Apart from clinical staging, molecular profiling enables targeting of the identified underlying alterations, rather than histology. In contrast to breast carcinoma, molecular classification of GC does not yet imply treatment modality. Molecular classifications of GC and their therapeutic implications are therefore extensively studied. The current proposed molecular divisions of GC come from three different parts of the world where different standard treatment modalities for advanced GC are recommended. Wider use of GC molecular subtyping may solve problems, such as susceptibility to novel systemic therapy regimens or selection of patients for aggressive surgery and targeted adjuvant/conversion therapy. In any case, the rapid entry of novel molecular targeted therapies into routine oncology practice clearly underscores the urgent need for clinicians to be aware of these new possibilities.

## INTRODUCTION

Gastric cancer (GC) continues to be one of the leading types of fatal cancer worldwide. Despite the ongoing decrease in morbidity and mortality it is still the fifth most common and the third cause of cancer-related death [1]. More than 70% of these cases occur in developing countries, especially in East Asia [2]. The majority of patients in the West are diagnosed with advanced disease, whereas early stage is found in only 6–10% of the cases. Therefore radical surgical treatment is frequently not possible in these patients with poor prognosis. The late diagnosis can be due to absence of significant symptoms at an early stage and the lack of validated screening programs. The expected 5-year survival rate for patients after surgery is approximately 26% in Western countries [3]. More than half of the curative operated GCs locally relapse or show distant metastases.

Systemic (chemo-)therapy is recommended for patients with stage II or III disease as perioperative (neoadjuvant) treatment followed by surgery, and for patients with stage IV as palliative single-modality treatment [4]. Chemotherapy can improve quality of life and prolong survival time, but patients with stage III and IV have 5-year overall survival (OS) rates of 9.2–19.8% and 4%, respectively [5]. Patients with metastatic GC generally receive only palliative chemotherapy, but a proportion of stage IV patients with good response to (induction) systemic therapy should be re-assessed with the intention to perform surgery [6]. There are many unresolved problems with this combined modality therapy of advanced gastric cancer: the question of the best perioperative and induction chemotherapy regimen; the issue of the selection of candidates for extended surgery who may benefit the most with clear definition of the goal of the surgery.

Different epidemiological and clinical features of GC are best reflected by the traditional Laurén histo-prognostic classification (intestinal, or diffuse type) which at least in the Western hemisphere is useful in clinical practice, being helpful to guide the extent of gastric resection for GC. Whether or not new classifications dividing GC into distinct molecular subtypes may contribute to define the strategy of more personalized onco-surgical therapy is not yet clear. In this review arguments will be provided to help clarify this issue.

## PATHOLOGY

Malignant tumors of the stomach include lymphomas, gastrointestinal stromal tumors, sarcomas and neuroendocrine tumors, but the most common malignancy is GC. Most GCs are adenocarcinomas, however, GC is a complex, heterogeneous and multifactorial disease with different phenotypes. This variety is the main cause of the existing diversity of histological classification systems. The most wildly used ones are the Laurén, the WHO, and the Goseki classification systems.

### Laurén classification

According to the Laurén classification, gastric adenocarcinomas are divided into intestinal, diffuse, mixed and indeterminate subtypes [4]. They vary not only in morphology but also in epidemiology, progression pattern, genetics and clinical picture. The most common subtype, the intestinal one, occurs in about 54% of the cases, it is twice as often in males as in females and is localized mostly in the antrum. Histopathologically, it is characterized by malignant epithelial cells that show cohesiveness and glandular differentiation infiltrating the surrounding tissue [7].

By contrast, the diffuse subtype (32%) is characterized by tumor cells that show poor differentiation and lack of cohesion. This subtype occurs equally often in males and females and these patients are on average younger than those with intestinal GC. Intestinal type of gastric cancer is felt to be caused mainly by environmental (exogenous) factors whereas the diffuse type is thought to be due to hereditary and genetic (endogenous) factors. The intestinal and diffuse GC subtypes are pathologically considered as separate entities, but clinically, both are treated similarly. The main clinical difference is related to the different recurrence patterns, with the diffuse-mixed types more prone to peritoneal dissemination, especially when the serosa is involved, whereas the risk of liver metastases is higher in the intestinal type. The prognostic relevance of Laurén classification remains controversial [8]. Nevertheless, in the last decades the overall incidence of GC has decreased, which is mostly contributed to the reduction in the rate of intestinal GCs. Presumably the most important reason of the diminished incidence of intestinal type of GC is the drop in *Helicobacter pylori (H.p.)* prevalence and the altered food habits. On the contrary, the relative incidence of the diffuse type GCs is increasing [9]. This difference is partly explained by difference in biology. In the intestinal type of stomach cancer, there is a well-established stepwise tumor progression model that provides a window for secondary prevention and early detection. Tumorigenesis of the diffuse type of stomach cancer is less well understood and there are as yet no well-defined precursor lesions. Not only the ratio between the types of stomach cancer shows a secular trend, but also the localization of tumors has changed over time. There is an increase in the incidence of gastric cardia and GE-junction cancer compared to distal cancers [10].

### WHO classification

Compared to the Laurén’s system, the WHO classification is based on pure histo-morphological appearance. The WHO divides GCs into tubular, papillary, mucinous, poorly cohesive (including signet ring cell carcinoma) and mixed carcinomas. This classification includes, besides adenocarcinomas, also all other types of gastric tumors [8]. When one compares the Laurén and the WHO classification tubular and papillary carcinomas fall within the intestinal type of stomach cancer, whereas signet-ring cell carcinoma and other poorly cohesive carcinomas correspond to the Laurén diffuse type [11].

### Goseki classification

The third mentioned scheme – the Goseki classification divides GC, based on intracellular mucin production and the degree of tubular differentiation, into four groups: group I: tubules well differentiated, intracellular mucin poor; group II: tubules well differentiated, intracellular mucin rich; group III: tubules poorly differentiated, intracellular mucin poor; group IV: tubules poorly differentiated, intracellular mucin rich. Most studies, which have focused on prognostic significance, did not confirm a prognostic independent value of this system [8].

Although current histopathological systems influence endoscopic or surgical choices, they are still insufficient to guide precision treatments for individual patients. Not only new therapies, but a new classification for GC is urgently needed as well.

### Precursor lesions for intestinal & diffuse subtypes (Correa cascade)

The multistep progression model of the intestinal GC is known as “the Correa cascade”. It starts with *H.p.*-related chronic gastritis which leads via gastric atrophy and paracancerous intestinal metaplasia to precancerous dysplasia [12]. As mentioned earlier, our insights into this multistep progression model have practical consequences for our therapeutic recommendations [13].

Unfortunately, the nature of diffuse gastric cancer remains far more elusive. In this case *H.p.*– induced chronic gastritis is also a risk factor and may well be a first, and in that case only recognised precursor lesion [14]. As further steps are proposed globoid dysplasia [15, 16] or signet-ring cell carcinoma *in situ* which precedes the evolution of Hereditary Diffuse Gastric Cancer (HDGC) [17]. But hereditary diffuse gastric cancer is an autosomal dominant disease caused by a germline mutation in the gene that encodes E-Cadherin and is not associated with *H.p.* gastritis.

In China, it was found that the incidence of gastric cancer at the population level was similar between participants receiving *H.p.* eradication treatment and those receiving placebo for over 7 years in a high-risk region. In the subgroup of *H.p.* carriers without precancerous lesions, eradication of *H.p.* significantly decreased the development of gastric cancer. Longer follow-up is needed to examine the effect of eradication in participants with precancerous lesions [18].

The globoid dysplasia or tubule neck dysplasia (TND) is characterised by architectural and immunohistochemical changes in the neck zone of the gastric pits or foveolae [19]. Foveolar cells (also known as mucus neck cells) which are located in the neck zone that forms the transition between the superficial gastric pits and the deeper glands with their specialized cells, transform into signet-ring cells [20]. These dysplastic cells are less cohesive due to the loss of E-cadherin once the second wild type allele has also lost its function due to the second hit. As a result, the isolated cells detach from the gland neck zone and further transform. This process has been described as “signet ring cell drippings” [21]. To this point the gastric mucosa remains intact. This is the stage of early GC, with the morphology of signet ring cell carcinoma (SRCC), which is described in the prophylactic gastrectomies of carriers of the E-cadherin germline mutation representing approximately 26% of early GCs [14]. Thereafter, in the natural course of the disease, the tumour grows, mutates and progresses to advanced diffuse GCs: Signet Ring Cell Carcinoma or poorly differentiated carcinoma (PDC) [22].

The above postulated steps in tumor progression of diffuse type gastric cancer are all based on our knowledge of hereditary diffused gastric cancer (HDGC), which is caused by a germline mutation in *CDH1* gene that encodes E-cadherin, and forms only maximally 3% of all GCs [17, 23]. One wonders whether it can be used as a model that is representative for all diffuse type gastric cancers. The triggers in the progression of the remaining of the diffuse subtypes (sporadic diffuse GCs) are still largely obscure. They may also have mutations within *CDH1*, but acquired as somatic alterations [24].

Single-nucleotide polymorphisms (SNPs), various acquired aberrations [e.g. chromosomal instability (CIN), microsatellite instability (MSI), other somatic gene mutations, and also epigenetic alterations may all contribute to tumorigenesis as well [25].

### Mucin characteristics

Mucins are the family of glycoproteins that form a protective gel layer on the surface of the mucosa [30]. One of them, MUC1, takes part in regulating cell homeostasis and therefore it has been categorized as oncoprotein [31]. The expression of mucins varies in different localisations of the digestive track and between healthy epithelium and histological subtypes of cancers. In GC the expression of typical mucins decreases with cancer progression [30]. Moreover, they are atypically changed either by alternative glycosylation or by dysregulation within the MUC genes [32]. Both quantitative and qualitative losses impair their functions.

The mucinous GC (MGC) and signet-ring cell carcinoma (SRCC) are two histologically different subtypes of GC which both present with an abundant number of mucin-derivatives [14]. In MGC extracellular mucin constitutes more than 50% of the tumour volume [33], whereas in SRCC at least 50% of its cells contain intracellular mucin-filled vacuoles [34]. MGC accounts for 2–6% of the gastric carcinomas, while SRCC for 3.4–39% [33]. Clinically, the presence of a mucinous component in GC relates to poor prognosis, likewise in colorectal cancer [33, 35] which is often related to an advanced and disseminated stage of the disease at the diagnosis [36, 37].

A healthy mucinous layer, which covers the surfaces of epithelial tissue, is strongly hydrophilic due to its biochemical structure. It is conceivable that changes in the mucin profile result in weakening the hydrophilic properties of the layer, which hinders cell penetration of well soluble derivatives of platin, or that in the high volume of extracellular mucin the cytotoxic agents dissolve in the gel before reaching cell membrane. These assumptions would be consistent with the general observation of poor effects of standard chemotherapy in mucinous and signet -ring gastric and colon cancers [38–40].

### EBV

The majority of GCs are associated with infectious pathogens such as *H. pylori* and Epstein-Barr virus (EBV). In EBV-positive GCs *PIK3CA* (80%), *ARID1A* (54%), and *BCOR* (23%) mutations were observed [26]. p110 alpha, a product of the *PIK3CA* gene, is a catalytic subunit of phosphatidylinositol 3-kinase (PI3K). PI3K signaling is involved, among others, in cell growth, proliferation, migration and survival. *ARID1A* regulates gene expression by chromatin remodeling. In turn, *BCOR,* Bcl-6 corepressor antiapoptotic protein, is associated with function and survival of some cells in the immune system. Moreover, somatic BCOR mutations have been confirmed in human cancers including retinoblastoma, medulloblastoma, osteosarcoma, and hepatocellular carcinoma. It is suggested that BCOR can also function as a tumor suppressor protein [41]. Some studies also showed a correlation between EBV infection and *JAK2*, *CD274*, *PDCD1LG2* and *ERBB2* amplification [2, 42]. The Janus kinase 2 (*JAK2*) gene plays a role in cell proliferation, survival and differentiation. Moreover, JAKs have an effect on the activity of PI3K/Akt/mTOR pathway [26]. *CD274,* also known as PDL1, and *PDCD1LG2* takes part in PD-1/PD-L1, PD-L2 pathway, which is involved in anti-tumor immune response. When PD-L1 binds to PD-1, an inhibitory signal is transmitted into the T cell. Tumor cells exploit this immune-checkpoint pathway as a mechanism to evade detection and inhibit the immune response. Expression of PD-1 or PD-L1 is associated with poorer prognosis in patients suffering from GC [26]. EBV-positive tumors are also characterized by a strong IL-12- mediated signaling signature, what suggests a robust response of immune cells [2]. *ERBB2*, also known as *HER2,* encodes a member of the epidermal growth factor receptor family. It activates the PI3K/Akt and Ras/MEK signaling pathway and plays a role in cell proliferation, adhesion, migration and differentiation. *HER2* amplification is related to worse prognosis and more aggressive disease [2]. DNA hypermethylation of promoter regions of tumor suppressor genes (i.e. *CDKN2A*) is another alteration, which is more common in EBV-positive than EBV-negative tumors [5, 11, 42].

### MMR deficiency

Inactivation of the DNA mismatch repair (MMR) machinery is responsible for microsatellite instability (MSI). GCs with MSI often show hypermethylation of the mutL homolog 1 (*MLH1*). The MLH1 protein, a product of the *MLH1* gene, plays an essential role in DNA mismatch repair and is responsible for fixing errors that occur during DNA replication. MSI-positive GCs are often associated with activation of the epidermal growth factor receptor (EGFR) and PI3K pathways [27].

### MSI (Microsatellite instability)

MSI is a genetic alteration consisting of repetitive microsatellites (short, repeated nucleotide sequences) caused by the loss of DNA mismatch repair activity [44].The role of MSI in GCs remains uncertain. In secondary post hoc analysis of the MAGIC trial GCs were classified as either microsatellite stable (MSS), or with low or high MSI, designated respectively as MSI-L and MSI-H. Patients with MSI-H tumors have superior survival compared with MSS/MSI-L tumors when treated with surgery alone. On the other hand, patients with MSI-H tumors have inferior survival when they were treated with chemotherapy plus surgery. Poor prognosis in MSI-H tumors was associated with neoadjuvant chemotherapy [45]. One hypothesis about the chemotherapy resistance is the presence, in MSI-H tumors, of a firm infiltrate of tumor infiltrating lymphocytes. Chemotherapy may have a negative influence on the hosts’ immunoresponse against the tumor and consequently may have an adverse effect on patients’ prognosis. Other studies suggested that prognostic value of MSI is limited to selected histotypes and location of tumors. Marrelli *et al.* indicated that MSI had impact on prognosis in non-cardia tumors of the intestinal type or tubular/poorly differentiated histology. Moreover, MSI-H status was more frequent observed in older patients, females and was associated with lower numbers of lymph nodes involved and lower chances of distant metastases [46]. Results similar to these data were also observed in other studies, where tumors with MSI were associated with lower propensity to nodal spread, intestinal histotype, distal location and better prognosis [47, 48]. Another meta-analysis also indicates that gastric cancer with MSI is associated with better overall survival as compared with MSS [49].The different proportion of cardia/non-cardia tumors and differences in histological types could explain inconclusive results regarding the prognostic and predictive role of MSI in different studies. Future studies checking the MSI status and necessity of perioperative Chemotherapy among those patients are needed.

### TP53

The most frequently mutated gene in GC is *TP53*. This mutation was observed in approximately 40% of GC cases [27]. TP53 is a tumor suppressor protein, which plays an important role in cell cycle arrest, metabolism, senescence, apoptosis and DNA repair. Moreover, the key role of TP53 - called ‘the guardian of the genome’ - is to maintain genomic stability [27].

### CIN and aneuploidy

The chromosomal instability (CIN) phenotype comprises altered DNA copy numbers (aneuploidy) and various changes in chromosome regions, such as translocation, amplification, deletion or the loss of heterozygosity (LOH) [25]. Aneuploidy may be the result of alterations such as defects in mitosis, tetraploidy and centrosomal abnormalities [29]. It must be noted, that CIN and aneuploidy are not equivalent but they are closely related in GCs. CIN tumors are frequently associated with activation of the RTK/RAS pathway and *EGFR, HER2, HER3, JAK2, FGFR2, MET, PIK3CA* and *KRAS/NRAS* amplification [28].

### Epigenetic alterations

As mentioned before, in some cases the inactivation of tumor suppressor genes or DNA repair genes in gastric cancer resulted from hypermethylation. Indeed, several studies have proved that epigenetic alterations affected the cancer-related genes (i.e. *APC, K-RAS, TP53, hMLH1, CDKN2A/p160*) even more commonly than genetic alterations (mutations) [43, 50, 52]. Hypermethylation associated with gene inactivation occurs at specific sites of the promoter sequences, defined as CpG islands [53]. Thus, the altered, tumor-specific gene hypermethylation is referred to as the CpG island methylator phenotype (CIMP). Tumors with multiple concurrently hypermethylated *loci* are described as high-CIMP. High-CIMP is frequently found in MSI-positive GCs, and is associated with hypermethylation of mismatch repair genes (hMLH1) [50]. The CIMP phenotype is thought to be an early event in gastric cancer. Its presence in adjacent normal tissue may be associated with *H. pylori* infection, which points to the possible mechanism of its contribution to GC tumorogenesis [50].

## MOLECULAR CLASSIFICATIONS OF GASTRIC CANCER

### Cancer genome atlas project

Recent studies have shown a correlation between molecular and clinico-pathological characteristics of GCs. The Cancer Genome Atlas Research Network Group (TCGA) proposed a molecular classification dividing GC into four subtypes: EBV-positive tumors, microsatellite unstable tumors (MSI), genomically stable tumors (GS) and tumors with chromosomal instability (CIN) [42]. Researchers studied GC tissue samples, which were obtained from 295 patients not treated with prior chemotherapy or radiotherapy. The samples were collected from institutions across the world. 107 tumor samples were characterized by array-based somatic copy number analysis, whole-exome sequencing, array-based DNA methylation profiling, mRNA sequencing, miRNA sequencing, reverse-phase protein array and by microsatellite instability testing and whole genome sequencing in comparison with the germline profile.

EBV activation was found in about 9% of the cases of GC. EBV-positive GCs were more prevalent in males (81%) and mainly localized in the gastric fundus and body (62%). MSI occurred in 22% cases of GC and was more frequent in females and older patients (median 72 years). The third group, GS, was defined by the lack of traceable molecular alterations and comprised about 20% of the GCs. These tumors were diagnosed at an earlier age and correlated with the Laurén diffuse type. GS cancers often showed mutations in genes responsible for cell adhesion, such as *RHOA*, *CDH1* and *CLDN18/ARHGAP*. GS subtypes also exhibited elevated expression of cell adhesion and angiogenesis-related pathways [28]. The last type of GC, defined as CIN, occurred in nearly 50% of the GCs and was associated with intestinal-type histology and localization in gastroesophageal junction and cardia [42]. The TCGA classification was not correlated with prognosis. It may however be useful in the selection of the preferred therapy.

### Asian cancer research group

Another classification was proposed by Asian Cancer Research Group (ACRG), who divided GCs into subtypes linked to distinct patterns of molecular alterations, disease progression and prognosis [54]. The samples were obtained from patients treated in a single referral hospital in South Korea and characterized by mRNA expression, somatic copy number and targeted gene sequencing. GCs were divided, similar as in the TCGA classification, into four groups: with microsatellite instability (MSI), microsatellite stable and epithelial-to-mesenchymal transition (MSS/EMT) phenotype, microsatellite stable and presence of TP53 (MSS/TP53+) or no TP53 signature (MSS/TP53-).

MSI subtype was correlated with the best prognosis and the lowest frequency of recurrence (mostly hepatic) of the GC subtypes. It was mostly diagnosed at early stages (I/II). This type was localized mainly in the antrum (75%) and in more than 60% of the cases it corresponded to Laurén’s intestinal subtype. MSI subtype was associated with the presence of hypermutation, with mutations in the genes *KRAS* (23.3%), *ALK* (16.3%) and *ARID1A* (44.2%), involved in the PI3K pathway (42%) and with loss of MLH1 expression.

The epithelial–mesenchymal transition (EMT) is a process by which epithelial cells lose their cell polarity and cell-cell adhesion. They gain migratory and invasive properties and acquire the phenotype of mesenchymal stem cells. In contrast to MSI, MSS/EMT GCs were diagnosed mainly in advanced stages (III-IV) and associated with a worse prognosis. What is more, they were associated with the highest recurrence frequency (63%) of the four subtypes, mostly peritoneal. EMT occurred in younger patients and corresponded, in about 80%, to Laurén’s diffuse type including a large set of signet ring cell carcinomas. This GC subtype showed loss of expression of the *CDH1* gene. EMT GCs had lower number of mutation events than other MSS subtypes.

The remaining group was further divided into two subtypes: *TP53* mutant and *TP53* wild type. MSS/TP53+ (26%) and MSS/TP53- (36%) had an intermediate prognosis (better in wt*TP53*) and also an intermediate chance of recurrence. MSS/TP53+ was frequently associated with EBV infection. MSS/TP53+ presented the highest prevalence of *TP53* mutations while the *TP53* wild type exhibited a higher frequency of mutations in genes such as *APC, ARID1A, KRAS, PIK3CA, SMAD4*. On the other hand, in MSS/TP53- subtype the genomic instability index (the number of altered chromosomes) was higher, and amplification of *HER2, EGFR, MYC, CCNE1, CCND1, MDM2, ROBO2, GATA6* and *MYC* both at the DNA and mRNA level was observed [54].

### Comparison of TCGA and ACRG classifications

TCGA and ACRG projects developed a four-group classification. Comparison of both classifications indicates the presence of similarities but also differences between them. MSI subtype was found in both classifications and characterized by high mutation frequency and the best prognosis. Afterwards, TCGA marked out also GS, EBV+ and CIN subtypes, while ACRG classification included MSS/EMT, MSS/TP53+ and MSS/TP53− GCs subtypes. When comparing the TCGA chromosomal unstable group and the ARCG MSS/TP53- group it is notable that TCGA chromosomally unstable subtype comprised a higher fraction of the total number of tumors than did the TP53 negative subgroup. CIN and GS TCGA tumors were present across all ACRG types. The difference in frequency of *CDH1* and *RHOA* mutations results in a TCGA GS class that is not equivalent to the ACRG MSS/EMT subtype [28]. Furthermore, the TCGA GS subtype is not equivalent with the ACRG MSS/EMT subtype either and the MSS/TP53+ group does not overlap with the TCGA EBV subtype. Possible reasons for these differences could be the larger proportion of Laurén diffuse type GCs among the ACRG group (24% in TCGA versus 45% in ACRG) and lesser numbers that were located proximally and at the GE junction. Also the ethnic origin of the patients was different (USA and Western Europe vs. Korea). Finally, different platforms for genetic studies in the two projects were used. Associations between genetic aberrations of cancer-related genes and clinical outcomes were studied in Korean GC patients. Researchers have compared both classifications, TCGA and ACRG, with patients outcomes. Analysis of clinical outcomes by using both classifications showed that EBV group had the best survival [55].

### ‘Singapore-Duke’ classification

Another project, the so called ‘Singapore-Duke’ study, was performed to identify subtypes of GCs with biological properties and sensitivity to particular chemotherapy and targeted agents [56]. The samples were obtained from 248 Singaporean and Australian patients. A comparison of gene expression patterns identified three major subtypes: proliferative, metabolic and mesenchymal.

The mesenchymal type had high activity of the epithelial-mesenchymal transition pathway. This subtype had high mRNA levels of *CDH2* and low levels of *CDH1*. High activities of cancer stem cell pathways were also found. It was associated with alterations in the transforming growth factor β, vascular endothelial growth factor, NFκB, mTOR, and sonic hedgehog pathways. *TP53* mutations were relatively uncommon. The mesenchymal type was also significantly enriched for tumors with low copy number alterations (CNA) and strongly associated with the Laurén diffuse type and poorly differentiated GCs. Cell lines of the mesenchymal subtype were particularly sensitive to phosphatidylinositol 3-kinase-AKT-mTOR inhibitors. This is consistent with the high activation of the mTOR pathway in these tumors.

The proliferative subtype was characterized by alterations of genes related to the cell cycle. It was associated with high activities of some oncogenic pathways, such as E2F, MYC and RAS. This type was strongly associated with the Laurén intestinal type and a low tumor grade. It was characterized by more frequent *TP53* mutations and more extensive copy number amplification. Those GCs were also associated with DNA hypomethylation, that may have a role in promoting chromosomal instability.

The third subtype, metabolic, showed a high activity of a pathway related to a particular kind of gastric metaplasia - spasmolytic-polypeptide-expressing metaplasia (=pseudopyloric metaplasia), which can be a step in the development of gastric adenocarcinoma. Cancer cells of these tumors were more sensitive to 5-fluorouracil than of the other subtypes. In each of the Singapore and Australian cohorts, patients with metabolic GC treated with 5-fluorouracil did better than those with surgery alone. It was also noted that Australian patients with mesenchymal GC treated with 5-fluorouracil had a better disease-free survival, but in the proliferative type there was no benefit from this treatment. In contrast, the Singapore patients with mesenchymal or proliferative subtypes had no benefits in cancer-specific or disease-free survival. The study also showed that the metabolic subtype cell lines were more sensitive than other cell lines for 5-fluorouracil. This sensitivity is probably associated with the significantly lower expression of thymidylate synthase (TS) and dihydropyrimidine dehydrogenase (DPD) in this subtype compared to the other two types. 5-fluorouracil activity is mainly dependent on inhibition of TS. A high level of TS is probably the reason for the 5-fluorouracil resistance. In addition, DPD participates in 5-fluorouracil degradation, so a high level of DPD presumably leads to rapid 5-fluorouracil degradation and tumor cell resistance for this cytostatic agent [56].

Analysis of cancer-specific survival showed no significant survival difference among the three subtypes. Only the proliferative-subtype patients had a worse disease-free survival in multivariate analysis. Higher TNM stages were associated with worse outcome.

### Simplified algorithm using immunohistochemical and *in situ* hybridization techniques

Advanced molecular techniques would be necessary to define subgroups of GCs, which is not feasible nor cost effective in every day practice. Setia *et al.* [57] proposed less expensive techniques that are available in routine diagnostic practice. They examined a cohort of 146 GCs Massachusetts using immunohistochemistry and *in situ* hybridization. On that basis they subdivided GCs into five groups according to EBV status, microsatellite instability, aberrant expression of E-cadherin and *TP53* status.

EBV-positive GCs were observed in 5% of the diagnosed cases and they were associated with better survival and membranous expression of PD-L1 in tumor cells. The expression of PD-L1 suggests a potential role for immunotherapy such as anti-PD-1/PD-L1 monoclonal antibodies. Microsatellite-unstable gastric cancers were characterized by better survival and lower frequency of lymph node metastasis. They were also associated with MUC2 expression and loss of MLH1 and PMS2. Gastric cancers with aberrant E-cadherin expression were related to the Laurén diffuse type. The frequency of this subtype correlated with the GS group in TCGA classification and the MS/EMT type of the ACRG. It was also comparable with the mesenchymal GC in the ‘Singapore-Duke’ study. Gastric cancers with aberrant TP53 expression were predominantly of the intestinal type. They were associated with a higher lymph node stage and increased HER2 expression. This subtype correlated with the CIN group in TCGA, the proliferative GCs in the ‘Singapore-Duke’ study and the MS/TP53- type in the ACRG. This subtype correlates with potential treatment responses upon inhibitors of receptor tyrosine kinases i.e., HER2, EGFR, VEGFR, c-MET, FGFR2 and cell cycle mediators such as CCNE1, CCND1 and CDK6 [51]. Gastric adenocarcinomas with wild type *TP53* expression have a gland-forming morphotype and increased expression of MUC6. This type corresponds with the metabolic GC described in the ‘Singapore-Duke’ study.

Ahn *et al.* [58] carried out a study in an Asian cohort (*n* = 349), where the same simple techniques such as immunohistochemical analysis and *in situ* hybridization were used. This project also classified GCs into five groups, on the basis of protein or mRNA expression of *MLH1*, E-cadherin, TP53, and EBV. EBV-positive tumors occurred mainly in the body of the stomach and were more frequent in males. The MSI subtype, that was characterized by aberrant *MLH1* expression, was associated with increased age, intestinal histology and antrum localization. Similarly, as in the previous study, EBV-positive and MSI tumors showed better overall survival. Tumors with aberrant E-cadherin expression were more common at younger age and correlated with advanced T and N stage and the worst overall survival. Cases with normal and aberrant TP53 expression showed an intermediate prognosis. Tumors with aberrant TP53 expression corresponded to the CIN group described by the TCGA or to MSS/TP53- described by the ACRG. Tumors with intact *TP53* activity overlapped with the MSS/T53+ group in ACRG and the metabolic class described by the Singapore-Duke group. The geographical differences were reflected in the prevalence of the subtypes that were observed in the various studies. The proportion of the MSI GC subtype was lower in the Asian cohort (7% vs 16% in the Western patients group), whereas the proportion of tumors with normal TP53 expression was higher in the Asian cohort (21% vs. 7%). Similar to the previous study, the GC subtypes proposed by Ahn *et al.* also corresponded with different relevant therapies [52]. Patients with the EBV-positive tumors, because of the *PIK3CA* mutations, *PD-L1*, *PD-L2* overexpression and *JAK2* amplification, may benefit from the treatment with *PIK3CA*, *PD-L1*, *PD-L2* or *JAK2* inhibitors. In the MSI subtype occasional mutations in *PIK3CA, ERBB2, ERBB3* and *EGFR* were identified. These lesions are thought to be targetable too. Tumors with *CDH1* and *ROHA* signaling pathways’ alterations seem to be more sensitive for S-1 plus cisplatin compared to 5-fluorouracyl plus cisplatin regimen. Tumors with functional loss of *TP53* were associated with alterations of many molecules such as *HER2, EGFR, VEGFR, c-MET, FGFR2*, which can be actionable targets. The remaining group, with normal TP53 activity was correlated *with APC, KRAS, ARID1A, PI3K and SMAD4* mutations. In this group an effectiveness of 5-fluorouracil treatment has been observed.

Knowledge of molecular background of GC may, in the future, help in determining the strategy of treatment and the use of personalized therapy. It remains very hard to predict responsiveness to therapy based on histopathological data alone. The classifications described above are, undoubtedly, an important step forward to the individualized treatment and development of the new molecular therapies.

## GASTRIC CANCER MOLECULAR TARGETS

### HER2

Human epidermal growth factor receptor type 2 (HER2) is a transmembrane tyrosine kinase and a member of the epidermal growth factor receptor family. It is involved in the regulation of cell proliferation, adhesion, migration and differentiation [26]. HER2 overexpression occurs in 7–34% GCs [2] and is more common in intestinal type and in gastro-esophageal junction (GEJ) tumors. HER2 positivity can be defined by protein expression on immunohistochemistry, by gene *ERBB2* amplification using fluorescence or chromogenic *in situ* hybridization (FISH or CISH) or Multiplex ligation probe amplification (MLPA). HER2 positive GCs can be successfully treated with anti-HER2 agents.

Trastuzumab, a recombinant humanized IgG1 antibody against the extracellular domain of HER2, blocks the HER2 receptor on the cell surface, sensitizes cancer cells to the tumor necrosis factor (TNF) and inhibits neoangiogenesis. Trastuzumab also shows anti-cancer activity by inducing antibody-dependent cytotoxicity [60]. As a result, this drug inhibits the proliferation, enhances apoptosis and reduces neoangiogenesis. The ToGA trial showed a survival benefit with the addition of trastuzumab to chemotherapy in HER2 positive GCs. This study became the basis for the approval of trastuzumab, in combination with capecitabine or 5-fluorouracil and cisplatin, for treatment of metastatic gastric cancer with HER2 overexpression [26].

Pertuzumab is another recombinant humanized monoclonal antibody directed against extracellular subdomain of the HER2 receptor. It should be emphasized that pertuzumab binds to a different HER2 epitope than trastuzumab. Due to the different interaction site of HER2, trastuzumab and pertuzumab act in a complementary manner, what has been demonstrated in HER2 amplified breast cancer treatment. The JACOB trial is an ongoing study, which aims to confirm the efficacy and safety of pertuzumab, combined with trastuzumab and cisplatin plus 5-flurouracil/capecitabine for patients with HER2-positive GC [2].

Lapatinib is the next agent, which blocks HER2 and EGFR-dependent signal transduction. However, the studies of its use have been disappointing. The LOGiC trial explored the efficacy and safety of CapeOx in combination with lapatinib or placebo as a first-line treatment for advanced or metastatic HER2-positive GCs. In turn, the TyTAN trial compared efficacy of paclitaxel alone or in combination with lapatinib as a second line regimen in patients with HER2-amplified advanced GC. Neither the LOGiC nor TyTAN trial showed survival benefits for patients treated with lapatinib [61, 62].

### VEGF

Vascular endothelial growth factor (VEGF) is one the most important mediators in tumor angiogenesis. VEGF increases the permeability of blood vessels, what leads to accumulation of fluid in the extravascular space and increases the pressure in the tumor tissue. As a consequence, fluid oppresses on the tumor cells and blood vessels, what hinders the entry of cytostatic agents into target cells [59]. In addition, VEGF stimulates neoangiogenesis and is a survival factor for cancer cells. Another function of VEGF is to increase activity of the fibrinolytic system in the tumor cells and in the extracellular matrix, what facilitates proteolysis of the surrounding tissue during angiogenesis. VEGF overexpression is a common feature in GCs and was found particularly in chromosomal instability (CIN) tumors. Treatment strategies targeting VEGF are based on using anti-VEGF monoclonal antibodies, tyrosine kinase inhibitors and VEGF receptor monoclonal antibodies [63].

Bevacizumab is a VEGF directed humanized monoclonal antibody. In the phase III clinical trial AVAGAST, bevacizumab in combination with 1st line palliative chemotherapy (based on capecitabine/cisplatin regimen) in advanced GC was evaluated. In the experimental arm no improvement on survival was noted. However, a subgroup analysis demonstrated longer OS for non-Asian patients [2]. Another phase III AVATAR trial, performed in China, evaluating the efficacy of adding bevacizumab to capecitabine/cisplatin regimen also did not show improvement on survival in patients with advanced GC [64]. UK Medical Research Council ST03 is a multicentre, open-label, randomised phase 2–3 trial conducted to investigate the impact of peri-operative chemotherapy with or without bevacizumab in operable oesophagogastric adenocarcinoma. The results of this trial also showed that the addition of bevacizumab to peri-operative chemotherapy did not improve overall survival in patients with potentially resectable oesophagogastric adenocarcinoma [65].

Apatinib is a small molecule tyrosine kinase inhibitor targeting VEGF-2. It has been tested in a phase III clinical trial, where GC patients who did not respond to second-line chemotherapy treatment were enrolled [66]. Both median PFS and OS were significantly improved in the apatinib group compared with placebo. Other small TKIs sunitinib and sorafenib in the ECOG5203 and STARGATE trials are currently under investigation [2, 13].

Ramucirumab is a humanized monoclonal antibody, which by blocking VEGF receptor-2 affects neoangiogenesis. Two trials, REGARD and RAINBOW showed that ramucirumab, either alone or in combination with paclitaxel, improved survival and disease control rate [67, 68]. Based on those studies the US Food and Drug Administration approved ramucirumab as a single agent or in combination with paclitaxel for the treatment of advanced GC that developed after fluorouracil platinum therapy [26].

### EGFR

The epidermal growth factor receptor (EGFR), also known as HER1, together with HER2, HER3 and HER4 belongs to the epidermal growth factor receptor family. Overexpression of EGFR occurs in 2.3–40% of the GCs and often indicates a poor prognosis. Treatment with the anti-EGFR monoclonal antibody turned out to provide a benefit in colorectal cancer [5]. Unfortunately, the current studies have not confirmed the efficacy of this treatment in patients with GC.

Cetuximab is a recombinant human-mouse chimeric EGFR IgG1 antibody, which when linked to its receptor leads to its degradation. In addition, cetuximab also has the ability to induce antibody dependent cell cytotoxicity [69]. The EXPAND trial, which evaluated the addition of cetuximab to the chemotherapy based on capecitabine and cisplatin in first line treatment of advanced GC, have not demonstrated improvements in progression-free survival (PFS). Moreover, the higher toxicity rate was noted [5].

Panitumumab is a humanized EGFR IgG2 monoclonal antibody. A REAL3 trial, where epirubicin, oxaliplatin and capecitabine with or without panitumumab in advanced GC were administered, showed inferior survival in the panitumumab group [70].

Also, other EGFR tyrosine kinase inhibitors such as gefitinib and erlotinib did not show survival benefit in GC [26].

Nimotuzumab is another kind of recombinant humanized monoclonal IgG1 antibody, which displays a cytotoxic effect in EGFR positive cancers. The efficiency of nimotuzumab and irinotecan combination as a second line treatment of advanced GC is currently tested in clinical trials (NCT01813253).

### FGFR2

The fibroblast growth factor receptor type 2 (FGFR2) has tyrosine kinase activity and plays a role in cell proliferation and angiogenesis. FGFR2 amplification occurs in 3–16% of the GCs and is associated with diffuse type and poorer prognosis [5].

Dovitinib is a multikinase inhibitor targeting FGFR, VEGFR, PDGFR, FLT-3, and other kinases. Studies evaluating efficacy and safety of dovitinib alone or in combination with chemotherapy are currently underway. Another FGFR inhibitor, brivanib, is also investigated in clinical trials [26].

Unfortunately, the SHINE trial, which proved clinical efficacy of selective FGFR1, 2 and 3 inhibitors - AZD4547 added to paclitaxel, failed to meet the primary endpoint - PFS [71].

### PI3K pathway

Activation of the PI3K/Akt pathway, associated with activating mutations of *PIK3CA,* monoallelic deletion of *PTEN* or *AKT* amplification, leads to increased cell proliferation and decreased apoptosis [7, 11]. Clinical trials evaluating the efficacy of PI3K inhibitors are currently underway and their results are awaited. Studies so far were without definitive clinical results.

Everolimus, an oral mTOR inhibitor, examined in the GRANITE-1 study, in monotherapy did not improve the survival of advanced GC patients compared to the best supportive care, though, an improvement in median progression free survival (PFS) was noted. Potential biomarkers, which can identify patients who would benefit from this treatment, are presently under identification. Furthermore, a randomized phase III trial evaluating the efficacy of everolimus in combination with paclitaxel in second line treatment is currently ongoing (NCT01248403) [26]. Treatment against tumors with a *PTEN* deficiency, which is an alteration often present in EBV-positive GC [42] , is under investigation (NCT01458067).

### MET

The mesenchymal-epithelial transition factor receptor (MET), which is activated by hepatocyte growth factor (HGF), is associated with promotion of gastric cancer cell proliferation, survival and migration [60]. MET amplification or overexpression, which occurs in 0–23% of the GC cases, can lead to activation of the deregulated MET pathway [5]. Anti-HGF/MET-targeted therapy is based on monoclonal antibodies, such as rilotumumab and onartuzumab, and tyrosine kinase inhibitors such as foretinib [60].

Rilotumumab is fully human monoclonal antibody directed against HGF. The RILOMET-1 study checking an efficiency and safeness of rilotumumab addition to the ECX (epirubicin/cisplatin/capecitabine) regimen in patients with HER2 -negative and MET-positive GC was terminated because of an increase in the number of deaths in the test arm compared to the control group [72].

Onartuzumab is a humanized anti-MET monoclonal antibody, which efficiency in the MET Gastric study was evaluated [73]. In this trial, treatment-naive patients with HER2-negative and MET-positive advanced GC were divided into two groups, receiving FOLFOX6 alone or with onartuzumab. Onartuzumab did not show an overall survival benefit [73]. Aside from MET, foretinib can inhibit other molecules, such as VEGFR-2, RON and ALK. Previous studies concerning the drug effect are not conclusive and there is a need for further studies [6, 11]. Studies of another MET kinase inhibitor - AMG337, despite the promising results of phase I trial, were halted during phase II, due to disappointing outcome [74].

### PD-1/PD-L1

The expression of PD-1 molecules on the T-cells surface is one of the immune tolerance mechanisms preventing their attack on own tissues. Cell activity against own tissues is inhibited by the binding of PD-1 molecules with their ligands PD-L1, which are also presented on the T-cells surface. PD1/PD-L1 interaction ensures that the immune system is activated only at the appropriate time in order to minimize the possibility of chronic autoimmune inflammation. When PD-L1 binds to PD-1, an inhibitory signal is transmitted into the T-cell, which reduces cytokine production and suppresses T-cell proliferation. Expression of PD-L1 was also detected on the tumor cells. These cells exploit the immune-checkpoint pathway as a mechanism to evade detection and inhibit the immune response. PD-L1 expressed on the tumor cells binds to PD-1 receptors on the activated T-cells, which leads to the inhibition of the cytotoxic T-cells. These deactivated T-cells remain inhibited in the tumor microenvironment. The PD1/PD-L1 pathway represents an adaptive immune resistance mechanism that is exerted by tumor cells in response to endogenous anti-tumor activity.

Studies showed that, among others, EBV-positive GCs in particular are characterized by an overexpression of both, PD-1 and PD-L1, what suggests that these tumors are a potential target for anti-PD-1 immunotherapy. A high PD-L1/PD-1 expression is associated with a significantly better patient outcome, and PD-L1 is an independent survival prognosticator. In patients with PD-L1-positive GC the immune checkpoint treatment may be used [75].Two monoclonal antibodies, pembrolizumab and nivolumab, are at present used in melanoma therapy. Their role in GC treatment needs to be investigated [71].Pembrolizumab, humanized anti-PD-1 antibody, was tested in the KEYNOTE-012 study; its IB phase demonstrated promising results with overall response rate 22% and median response duration of 24 weeks [5]. Nivolumab is being tested in a phase III trial in advanced, previously treated GC. In addition, its efficacy is being evaluated in combination with CTLA-4 blocker - ipilimumab in phase Ib/II study in patients with metastatic GC (NCT01928394) [5]. The results of those trials are awaited. A study by Le and colleagues showed that cancers with higher level of somatic mutations show a higher response rate to PD-1/PD-L1 inhibition [76]. Phase 3 study, NCT02267343, examined the efficacy and safety of nivolumab in patients with advanced gastric or gastro-oesophageal junction cancer after treatment with two or more chemotherapy regimens. The survival benefits indicate that monoclonal antibody inhibitor of programmed death-1 might be a new treatment option for that group of patients [77]. As gastric cancer, together with melanoma and lung cancer, belong to this group, and the benefits of immunotherapy are confirmed in the latter tumors, GC may also be suitable target for anti-PD-1 treatment.

Results of various targeted therapies for GC are presented in Table 1.

**Table 1:** Targeted therapies for gastric cancer

Trial	Target	Agent	OS benefit
**ToGA**	HER2	trastuzumab	**Yes,** 1st line, fluoropyrimidine + cisplatinum +/-trastuzumab, 13.8 vs 11.1 months
**EXPAND**	EGFR	cetuximab	No
**REAL-3**	EGFR	panitumumab	No
**TyTAN**	EGFR/HER2	lapatinib	No, 2nd line
**AVAGAST**	VEGF	bevacizumab	No, Yes, in non-Asian patients
**REGARD** **RAINBOW**	VEGFR-2	ramucirumab	**Yes**, 2nd line, ramucirumab vs BSC in advanced GC, 5.2 vs 3.8 months **Yes**, 2nd line, paclitaxel +/− ramucirumab, 9.6 vs 7.4 months
**GRANITE-1**	mTOR	everolimus	No, 2nd line
**1b KEYNOTE-012** **phase I study**	PD-1	pembrolizumab	Ongoing; manageable toxicity profile and promising antitumor activity
**ONO-4538-12** **phase III study**	PD-1	nivolumab	Ongoing; improved survival in previously treated advanced GC (median OS 5.32 months vs 4.14 with placebo)

OS benefit - overall survival benefit, HER2 - human epidermal growth factor receptor type 2, EGFR - epidermal growth factor receptor, VEGF - vascular endothelial growth factor, VEGFR-2 - vascular endothelial growth factor receptor-2, mTOR - mammalian target of rapamycin.

These data show that despite using targeted therapy there is no guarantee of its effectiveness. It may be related to intratumoral heterogeneity and sampling errors, which can lead to false negative and false positive results of tests that identify predictive molecular lesions. Moreover, reliable predictive biomarkers of response to a particular treatment are still missing. Many clinical trials were performed on unselected groups of patients. Moreover, at present, except HER2, there are no standardized criteria to evaluate gene amplification/overexpression [28] in gastric cancer. The molecular profiling of a single tumor biopsy sample may not be sufficient to guide targeted therapy but the issue of the number of tissue samples, which should be tested remains unresolved [71].

### Clinical aspects

Surgical resection of gastric cancer is potentially curative. Since the majority of patients relapse following resection alone, combined modality therapy is standard for non-early (advanced; ≥ stage IB) disease. For patients who have undergone upfront surgery without administration of preoperative chemotherapy, postoperative adjuvant radio- and/or chemotherapy is recommended. Patients with metastatic GC generally receive only palliative chemotherapy, but a proportion of stage IV patients with good response to (induction) systemic therapy should be re-assessed with the intention to perform surgery [5]. However, an extent of such surgery (lymphadenectomy, metastasectomy, multivisceral resection, or reduction gastrectomy) remains a subject of debate. This aggressive approach was defined as conversion therapy with surgery aimed to achieve an R0 resection, when metastases that were unresectable or marginally resectable (for technical and/or oncological reasons) had been controllable by chemotherapy. The new biological categories of stage IV GC classification, which are defined based on onco-surgical treatment strategies has been proposed [78].

The incidence of adenocarcinoma of the esophagogastric junction (EGJ) has increased in Europe and North America during the last 50 years, while more distal GC declined over the same time period [79]. The revised gastric cancer staging system applies to tumours arising in the more distal stomach and to tumours arising in the proximal 5 cm but not crossing the EGJ [80]. Those esophageal cancers crossing the EGJ, as well as proximal and distal gastric cancers are biologically different entities. It is speculated whether a shorter than 5 cm proximal resection margin might suffice in the context of transhiatal total gastrectomy for EGJ tumours [81, 82]. Thus surgery may be individualized to achieve proximal margin clearance. Since diffuse type pathology has become relatively more prevalent in the Western hemisphere, it would be important to predict the risk of proximal oesophageal involvement in that specific subset of EGJ tumours. This was shown to be possible by evaluation of E-cadherin expression on immunohistochemistry of the primary tumour sample [83]. Using the principle of the 2 cm proximal resection margin for resections performed with curative intent for true carcinoma of the gastric cardia (Siewert type II), the proximal extension of the resection (margin) was significantly shorter in E-cadherin negative tumours than in E-cadherin positive tumours. Histological type and stage of the tumour, lymph node metastases, and absence of E-cadherin expression correlated with proximal resection line involvement. However, only the absence of E-cadherin expression appeared to be a significant independent predictor of proximal resection line involvement.

General outcome of GC patients with distant metastases remains poor and palliative systemic therapy is recommended as a standard treatment, as established in the recently published REGATTA trial, which showed no survival benefit from limited surgery in comparison with chemotherapy alone in patients with one incurable organ site of metastases [84]. However, according to the National Comprehensive Cancer Network (NCCN) and Japanese Gastric Cancer Association (JGCA), in symptomatic stage IV GC patients with bleeding or obstructing tumours surgery can be considered as a palliative treatment in order to improve the poor quality of life [85]. On the other hand, ESMO guidelines consider both gastrectomy and metastasectomy as an experimental treatment until further evidence from randomized controlled trials is shown. Results of the randomized GYMSSA trial compares gastrectomy with metastasectomy followed by systemic treatment versus systemic therapy alone in advanced GC are therefore highly anticipated [86]. Nevertheless, there are reports presenting benefits from aggressive surgical treatment of liver metastases from GC. A meta-analysis published by Petrelli *et al.* evaluated long-term outcomes of 870 patients who underwent resection of liver metastases from GC [87]. This review showed that multiple and large metastases are associated with poor prognosis, whereas appropriate selection of patients for surgery of metachronous lesions may provide a satisfactory 5-year overall survival. The study revealed a significantly higher survival rate in the group of patients who underwent the most aggressive local treatment for hepatic metastases in comparison with patients who underwent only palliation or systemic treatment. Furthermore, palliative local treatment of liver metastases from GC had a higher survival rate when compared to palliation without local treatment of liver metastases. These results correspond with pooled-analysis by Markar *et al.*, which included 991 patients undergoing hepatic resection for liver metastases from GC [88]. This systematic review reported 1-, 3-, and 5-year survival rates of 68%, 31%, and 27%, respectively, with a median survival of 21 months in patients undergoing surgical resection of liver metastases from GC. Absence of dissemination, good control of primary gastric tumour, ability to achieve margin-negative hepatic resection, as well as solitary hepatic metastases had the best prognosis in this meta-analysis. Virtually no long-term survival (5-year OS: 0–2,1%) may be expected after palliative surgical approach without hepatic resection, whereas wide-ranging proportion (9, 3%–60%) of selected patients is still alive 5 years after aggressive surgical approach removing all visible disease [89].

For GC patients who develop peritoneal metastases (PM), an aggressive, multimodal treatment based on complete cytoreductive surgery (CRS) with hyperthermic intraperitoneal chemotherapy (HIPEC) have changed the so far dismal prognosis [90]. Clinical outcome in several studies shows improved survival in patients undergoing CRS and HIPEC in comparison with gastrectomy or palliative chemotherapy alone [91]. Moreover, completeness of cytoreduction is to be considered as an independent prognostic factor in several data, showing a significantly greater 1-, 3- and 5-year survival. However, even though an improved survival might be achieved, CRS and HIPEC cannot be considered as curative treatment [92, 93].

Although recommendations for adjuvant treatment of gastric cancer are based on the same scientific publications, different guidelines in various parts of the world are respected.

Data from Spanish the AGAMENON National Cancer Registry of patients with GC and EGJ HER2-negative tumours were used to test chemosensitivity of various Laurén types [94]. The chemotherapy regimens, based on two-agents with cisplatin or oxaliplatin and fluoropyrimidine, two or three agents with irinotecan, three agents with anthracyclines and docetaxel, were analyzed. The end points were OS, progression-free survival (PFS) and overall response rate (ORR). Diffuse type tumors were found to be less chemosensitive and associated with increased mortality. There was no preferred schedule in this type of GC. In intestinal type anthracycline and docetaxel based regimens increased ORR and docetaxel schedules increased OS. Therefore, Laurén classification of GC seems to be correlated with survival prediction and response to chemotherapy.

## CONCLUSIONS

The molecular division of GC comes from three various parts of the world. Similarly there are also three different standard treatment modalities for advanced gastric carcinoma combining local (surgery and radiotherapy) and systemic treatments. So far the molecular classification of GC does not imply modality treatment, like in breast carcinoma. We may speculate that some special molecular types of GC will be treated in different way than that suggested by current clinical guidelines [28, 71].
